# Bean and Pea Plastoglobules Change in Response to Chilling Stress

**DOI:** 10.3390/ijms222111895

**Published:** 2021-11-02

**Authors:** Joanna Wójtowicz, Joanna Grzyb, Joanna Szach, Radosław Mazur, Katarzyna B. Gieczewska

**Affiliations:** 1 Department of Plant Anatomy and Cytology, Faculty of Biology, Institute of Experimental Plant Biology and Biotechnology, University of Warsaw, I. Miecznikowa 1, PL-02096 Warsaw, Poland; j.wojtowicz@biol.uw.edu.pl (J.W.); j.skupien@biol.uw.edu.pl (J.S.); 2 Department of Biophysics, Faculty of Biotechnology, University of Wrocław, F. Joliot-Curie Street 14a, PL-50383 Wrocław, Poland; joanna.grzyb@uwr.edu.pl; 3 Department of Metabolic Regulation, Faculty of Biology, Institute of Biochemistry, University of Warsaw, I. Miecznikowa 1, PL-02096 Warsaw, Poland; rmazur@uw.edu.pl

**Keywords:** plastoglobules, bean, pea, chilling stress, chilling tolerant plant, chilling sensitive plant

## Abstract

Plastoglobules (PGs) might be characterised as microdomains of the thylakoid membrane that serve as a platform to recruit proteins and metabolites in their spatial proximity in order to facilitate metabolic channelling or signal transduction. This study provides new insight into changes in PGs isolated from two plant species with different responses to chilling stress, namely chilling-tolerant pea (*Pisum sativum*) and chilling-sensitive bean (*Phaseolus coccineus*). Using multiple analytical methods, such as high-performance liquid chromatography and visualisation techniques including transmission electron microscopy and atomic force microscopy, we determined changes in PGs’ biochemical and biophysical characteristics as a function of chilling stress. Some of the observed alterations occurred in both studied plant species, such as increased particle size and plastoquinone-9 content, while others were more typical of a particular type of response to chilling stress. Additionally, PGs of first green leaves were examined to highlight differences at this stage of development. Observed changes appear to be a dynamic response to the demands of photosynthetic membranes under stress conditions.

## 1. Introduction

Plastoglobules (PGs) are lipoprotein particles present in various plastid types such as proplastids, chloroplasts and gerontoplasts [1]. The diameter of these structures ranges from 30 nm to 5 µm and varies in different species and plastid types [1,2]. Chloroplast PGs are physically attached to the thylakoid membrane in the outer half of the thylakoid’s lipid bilayer, which surrounds the plastoglobules. The tight relationship between PGs and thylakoids enables an interchange of metabolites such as lipids and proteins [3,4].

Plastoglobules are mainly composed of neutral lipids (triacylglycerols, free fatty acids), prenyl quinones (α-tocopherol, plastoquinone) and carotenoids [5]. The highly hydrophobic PG interior is enclosed by a membrane lipid monolayer studded with proteins. The plastoglobule proteome of *Arabidopsis thaliana* consists of seven structural proteins from the PAP/fibrillins family (FBN1a, FBN1b, FBN4, FBN7a, FBN7b and FBN8), which provide a protein coating and prevent coalescence of the PGs. An additional 30 proteins are stated to be involved in the metabolism of isoprenoid-derived molecules (quinones and tocochromanols), lipids, and carotenoid cleavage [1,6,7,8].

Presented in the past as passive lipid droplets, plastoglobules have turned out to be the site of crucial metabolic pathways such as those leading to phylloquinone [9,10], tocopherol [9], carotenoids [7] and jasmonic acid (JA) biosynthesis [11], as well as involved in plants’ response to stress by accumulating antioxidants (e.g., plastochromanol-8, α-tocopherol) and sequestrating toxic molecules (e.g., fatty acid phytyl esters) [1].

In chloroplasts, PGs are associated with thylakoid membranes [3,12], which suggests that they play a dynamic role in thylakoid membrane function [13]. Variation in PG size and number in chloroplasts depends on the fitness of thylakoid membranes and their developmental stage [1]. Indeed, plastoglobules enlarge during thylakoid disassembly in senescing chloroplasts [14,15], acting as a reservoir for thylakoid membrane lipids. Their accumulation in senescing rosette leaves of *Arabidopsis thaliana* correlates temporally with the activation of diacylglicerate acyltransferase 1 (AtDGAT1) and with enhanced synthesis of triacylglycerols (TAG) [16]. Furthermore, plastoglobules are involved in the formation of thylakoid membranes in de-etiolated plastids [17]. After exposure to light, the number of plastoglobules decreased in barley etioplasts as prolamellar bodies were converted into thylakoid lamellae [18]. Plastoglobules are also implicated in chloroplast to chromoplast transition, where PGs enlarge and accumulate extensive amounts of esterified isoprenoids [5].

Furthermore, recent findings suggest that PGs actively participate in abiotic stress responses due to their dynamic role in various metabolic pathways and plastid development [19]. Adverse conditions such as drought, high salinity or high light intensity lead to the formation of reactive oxygen species, most significantly in chloroplasts. Accumulation of ROS causes oxidative damage, including lipid peroxidation, denaturation of proteins, and damage to nucleic acids. Controlling redox homeostasis is therefore essential for maintaining an active metabolism. Lipophilic antioxidants such as xanthophylls and prenylquinones (α-tocopherol, plastochromanol-8, plastoquinone-9) are present in chloroplasts and effectively scavenge ROS [20]. Tocopherols have been detected in all chloroplast membranes, most notably in plastoglobules [20,21], where almost 50% of the chloroplastic tocopherol pool is accumulated, representing 25-fold enrichment concerning the thylakoids [22]. Tocopherols act as membrane protectors against ROS [21] and prevent photosystem II from being photoinactivated [23]. The key enzyme of tocopherol synthesis—VTE1, the tocopherol cyclase—is located in the plastoglobule membrane [9]. In PGs’ interiors, VTE1 participates in tocopherol recycling and in another chloroplastic antioxidant representative’s biosynthesis—plastochromanol-8. The absence of tocopherols is accompanied by a lowered VTE1 level, which causes increased photoinhibition under photo-oxidative stress [6,24] and a chilling-sensitive phenotype [24,25]. Moreover, tocopherol deficiency in stress conditions plays a role in cellular signalling by altering jasmonic acid biosynthesis [26]. JA signalling is considered strictly involved with adapting plants to cold stress [27]. In addition, the first stages of the JA pathway are believed to be associated with plastoglobules, as four enzymes involved in this process temporarily localise in PGs [28].

Plants are often subjected to different environmental stresses. Chilling stress, i.e., the ability of a plant to tolerate low temperatures in the range 0–10 °C, is one of the most critical environmental factors affecting plant growth and limiting the growth and yield of many agricultural vital plants. Plants differ in their ability to adapt to cold temperatures. Some, such as beans, are considered chilling-sensitive (CS) plants and others, such as peas, chilling-tolerant (CT) ones. Pea and bean plants represent a perfect model for studying the different responses to chilling stress. Both plant species play an essential role in sustainable agriculture, both for human food and animal feed. Both species increase food production due to their ability to bind atmospheric nitrogen because of symbiosis with rhizobial bacteria. However, the production of these species is very often limited by different abiotic stresses, among them chilling stress. We chose these two species for our experiments because we have already examined their photosynthetic apparati [29,30,31,32]. From our previous papers [29,30], we learned that pea and bean chloroplasts differ in the arrangement of appressed and non-appressed thylakoids, even though these two plant species have very similar requirements for light and nutrition conditions.

There is evidence that plastoglobules act as a platform for antioxidant and JA biosynthesis, which seems altered in distinct plastid developmental stages or various abiotic stress conditions, including chilling stress. It is also interesting to study PGs from the first green leaves compared to mature ones and notice when the specific composition of these particles is defined.

The present paper examines plastoglobules isolated from first and mature leaves of two plant species with different chilling sensitivities—pea (*Pisum sativum*) and bean (*Phaseolus coccineus*)—both in control conditions and under chilling stress conditions commonly observed in nature. We demonstrate changes in the structural, molecular and physical properties of PGs due to applied stress conditions and plastid development phases. Furthermore, we provide evidence that plastoglobules play an active role in the response to chilling stress.

## 2. Results

### 2.1. Visualisation of PGs

#### 2.1.1. Transmission Electron Microscopy

**Figure 1:**
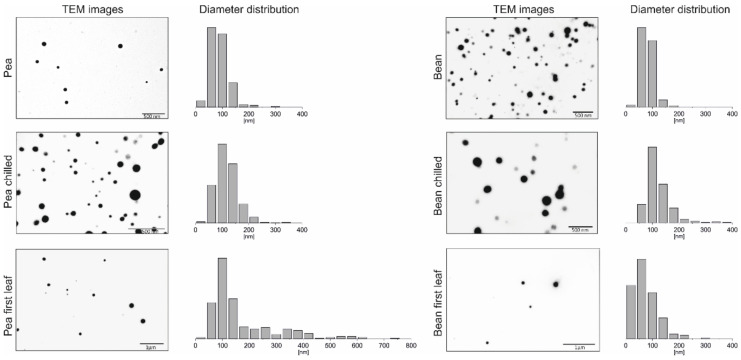
Visualisation and size distribution of plastoglobules (PGs) isolated from pea and bean first leaves and mature plants grown in control conditions and after the seventh day of chilling stress treatment. Isolated PGs were presented in electron micrographs obtained by transmission electron microscopy (TEM). The diameter of the visualised PGs structure was measured using the DigitalMicrograph 3.4 software (Gatan Inc., Pleasanton, CA, USA) and demonstrated on histogram charts; Y-axis represents % of size share in total counts; for total number of counts see Figure S1.

PGs, as lipid-rich plastid particles, can be easily stained by osmium tetroxide (OsO_4_) and visualised using transmission electron microscopy (TEM). It was previously demonstrated that their size varies depending on the species, development stage or plastid type [1]. Simultaneously, PG lipid-to-protein ratios result in their diverse osmiophilicity (staining intensity) [1,12,33]. The presence of isolated PGs in this paper was confirmed by TEM imaging. The demonstrated electron micrographs show round, opaque PGs unequally spread on the copper mesh surface (Figure 1).

The size of PGs ranged widely from 20 to approximately 720 nm, peaking between 50–150 nm in both analysed plants, which is consistent with earlier reports [12]. However, an additional population of more immense structures in pea plants was observed, not visible in CS plants, with a diameter ranging between 300 to 350 nm (Figure 1). After chilling stress treatment, both plants exhibited an increase in PG size, especially in the range of 150–270 nm. The most significant differences captured by TEM imaging were observed in PGs isolated from pea and bean first leaves, compared to those of mature leaves (Figure 1). Pea first leaf PGs displayed a wide diameter distribution, varying in size from 20 to 720 nm, resulting in the largest detected PG. In comparison, bean first leaves were revealed to possess smaller PGs than chilled bean plants, yet larger than those identified in plants grown in control conditions.

#### 2.1.2. Atomic Force Microscopy

**Figure 2:**
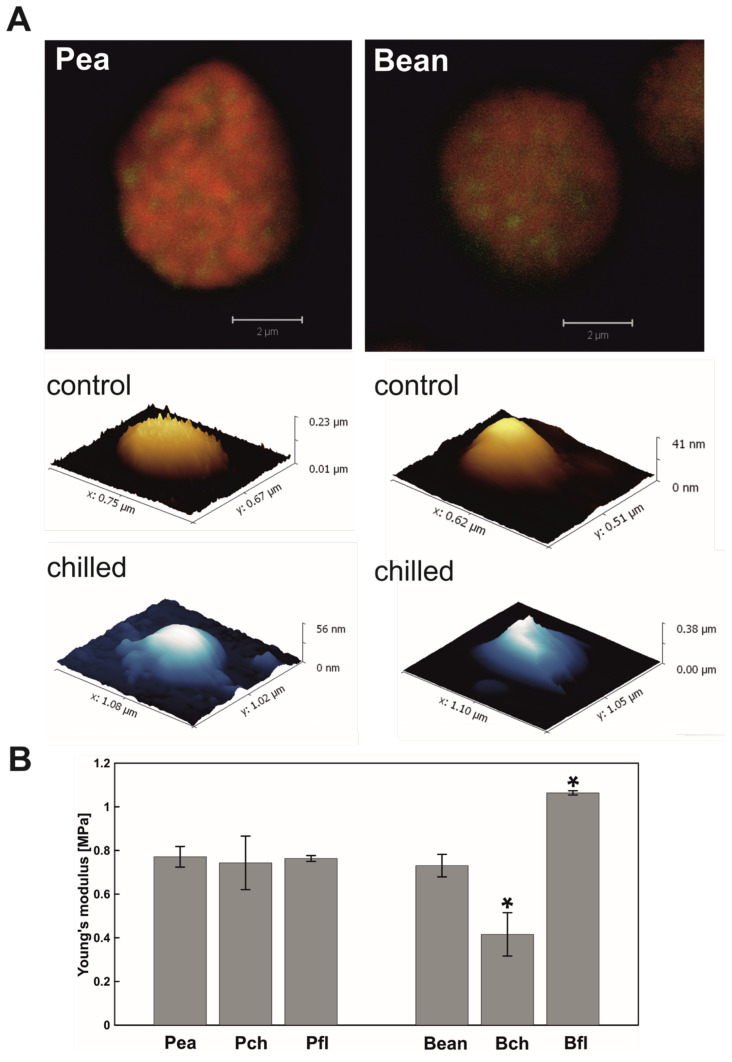
Characterisation of plastoglobules (PGs) from pea and bean plants: (**A**) localisation of PGs (green) in chloroplasts (red) observed with a CLSM (Confocal Laser Scanning Microscope), and topology of PGs in control conditions (yellow-to-white colouring) and after seventh day of low-temperature treatment (blue-to-white colouring) using AFM techniques; (**B**) elasticity of isolated PGs measured via Young’s modulus using AFM from mature plants in control conditions (Pea, Bean), after seven days of low-temperature treatment (ch), and in first leaves (fl). Presented data are mean values ± SD from three independent experiments; pairs of results marked with an asterisk differ significantly at *p* = 0.05 (one-way ANOVA with posthoc Tukey test).

PGs, as imaged with atomic force microscopy (AFM), were spherical, soft structures. The elasticity of all tested PGs (Figure 2B), measured by Young’s modulus (*E*), was close to 1 MPa. This value is in the range of fibroblasts and structures like gelatin [34] and significantly lower than the elasticity of collagen [35]. The specific *E*, however, differed between our groups. Pea PGs were almost the same from the point of view of elasticity (Figure 2B). However, when chilling was applied to the bean PGs, *E* decreased, meaning that the structure became softer than in control conditions. PGs isolated from bean first leaves were almost 25% more rigid than those in pea first leaves.

#### 2.1.3. FTIR (Fourier Transform Infrared Spectroscopy)

**Figure 3:**
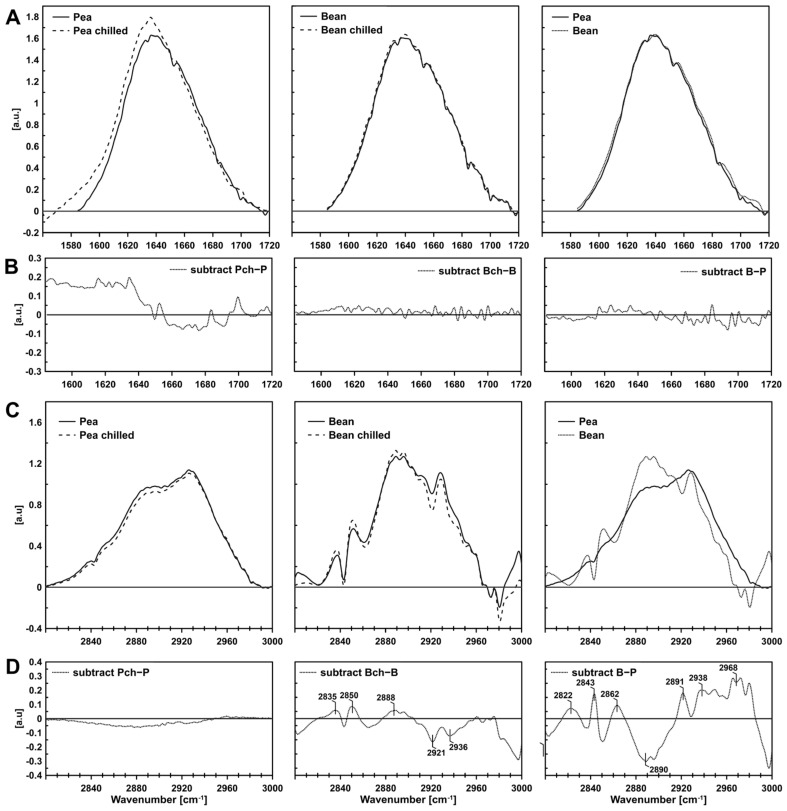
Representative spectra of infrared spectroscopy: the Amide I region (**A**) and its subtracts (**B**), the lipid acyl chains region (**C**) and its subtracts (**D**).

The FTIR method was used to analyse membrane physical properties (membrane fluidity) of isolated PGs to determine plastoglobules’ specificity—their composition and physical properties, and possible interactions between proteins and lipids within the structure. The inference was based on a detailed analysis of absorption bands, selectively corresponding to the vibrations of methyl and methylene groups of fatty acids, the C-H band at 3000–2800 cm^−1^, and the vibrations of the carbonyl group of a peptide bond, the Amide I band at 1710–1585 cm^−1^. Samples were measured using the ATR (Attenuated Total Reflection) method, designed to equalise the sample’s signal throughout the defined penetration depth into the sample. As a result, the most significant differences were observed between species rather than between the control plants and those subject to chilling stress (Figure 3).

The FTIR spectra of biological systems, such as thylakoids or plastoglobules, are complex due to the main molecules’ overlapping absorption [36]. When analysing the stretching vibration bands of the CH bonds of the 3000–2800 cm^−1^ methyl group, the following types of stretching vibrations are distinguished: asymmetric and symmetrical bonds of the methylene group (near 2920 cm^−1^ and 2850 cm^−1^) and symmetrical CH_2_ groups of lipids stiffened by interaction with transmembrane helices (the so-called boundary lipids) (2825–35 cm^−1^) [37]. The other components were asymmetric vibrations of the CH_3_ group near 2960 cm^−1^ and symmetric ones near 2870 cm^−1^. A slight increase in the boundary lipid component (Figure 3D, 2835 cm^−1^) was observed in bean PGs after chilling, and in bean compared to pea PGs.

However, the most significant difference between pea and bean PGs was the component 2890 cm^−1^ detected approximately 30% lower in bean PGs. The region of symmetric CH_2_ vibrations (2890 cm^−1^) can testify to the amount of unsaturated fatty chains in membrane lipids [37,38]. The lower the content of this component, the more saturated the fatty acid chains and the more rigid the membrane. At the same time, the component at approximately 2960 cm^−1^ was higher in bean PGs. This component is characteristic of asymmetric vibrations of the CH_3_ group and may indicate a higher content of shorter fatty acid chains [37]. In theory, the shorter the chain, the greater the fluidity of the membrane. Thus, the observed differences may balance and complement each other to give the desired shape and fluidity to the bean PGs in one leaflet of the membrane.

### 2.2. Composition of PGs

#### 2.2.1. Plastoglobulin Content

**Figure 4:**
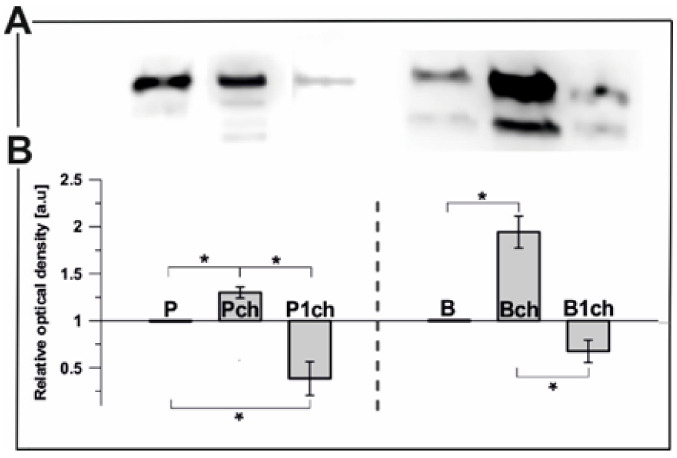
The relative optical density of the FBN1a protein (PGL35) in plastoglobule fractions extracted from pea (P) and bean (B). The data presented in the graphs are based on the immunodetection of electrophoretically separated proteins (**A**) of isolated plastoglobules from plants grown under chilling stress conditions—after the first (1ch) and seventh (ch) day of low-temperature treatment in comparison to control plants (**B**). Presented data are mean values ± SD from 3 independent experiments; pairs of results marked with an asterisk differ significantly at *p* = 0.05 (one-way ANOVA with post-hoc Tukey test).

PGL35 (FBN1a) is one of the most abundant plastoglobule structural proteins that falls under the fibrillin family (FBNs) and is bound by the membrane lipid monolayer which surrounds those lipoprotein particles [1]. The use of antibodies for immunoblotting resulted in detection and protein level quantitation in PGs isolated from all analysed plants (Figure 4). In the control plants, the content of PGL35 in pea PGs was 66% higher than in bean PGs (Figure 4A; P to B ratio = 1.66). Surprisingly, no sign of PGL35 was detected in either bean or pea first leaf PGs (not shown). The protein levels of both pea and bean structures after one day of chilling stress were reduced. Compared to control conditions, long-term temperature stress increased PGL35 abundance in Bch and Pch PGs (Figure 4). Moreover, obtained results showed the presence of two distinct bands with a predicted molecular mass of 35 KDa parallel to FBN1a, the second one marginally lower and probably corresponding to the distinct fibrillin FBN1b, as previously reported [39,40,41].

#### 2.2.2. Transcriptional Analysis

**Figure 5:**
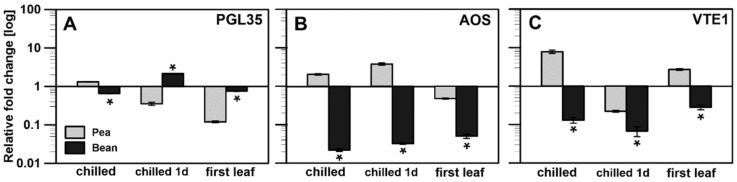
Transcript levels of genes: PGL35 (**A**), AOS (**B**) and VTE1 (**C**) in pea and bean normalised to 1 in control conditions; chilled: after seven days of chilling treatment, chilled 1d: after first night of chilling stress and first green leaves after three days of development in light. The data are mean values ± SD from three independent experiments; bean results marked with an asterisk (*) differ significantly at *p* = 0.05 from corresponding pea results in equal growth conditions/stage of growth.

Previous research showed that exposing plants to abiotic stresses (high-light stress, drought) affects plastoglobules’ size and content [6,42,43]. This situation may involve specific functions of proteins localised in or associated with PGs, as previously debated [4,6,11,44]. Therefore, the transcript levels of three genes were examined, which seemed to be the most promising method in the literature for describing PGs compositions. The levels of plastoglobulin (PGL35), allene oxide synthase (AOS) and tocopherol cyclase (VTE1) are depicted in Figure 5.

The PGL35 transcript levels were similar in both plants in control conditions (not shown), whereas the PGL35 protein level was lower in the bean plant (Figure 4A). An increase was observed in bean after a short chilling treatment (Figure 5A), as opposed to downregulation of PGL35 in pea. A longer chilling period equalised the transcript level for both plants. The most significant downshift in the PGL35 transcript level was noted in pea first leaves (Figure 5A).

AOS is a crucial enzyme in oxylipin biosynthesis (including jasmonic acid) involved in plant responses to various stress conditions, including chilling stress [45]. AOS transcript levels were markedly decreased in all stages of the experiment in both bean first and mature leaves, while in pea, an increase in the transcript accumulation of the gene under stress conditions was observed (Figure 5B).

Finally, the most extensive changes were observed in the tocopherol cyclase transcript level (Figure 5C). Under control conditions, pea and bean transcript levels were parallel, whereas, after chilling treatment, different strategies were applied. While pea samples were characterised by an increase in the transcript level, both in first and mature leaves under stress (Figure 5C: chilled, first leaf), in each of these cases, the transcript level in bean was lowered several-fold. Only after the first day of chilling was the transcript level of VTE1 lower in both plants.

#### 2.2.3. Lipid Enrichment of PGs

**Figure 6:**
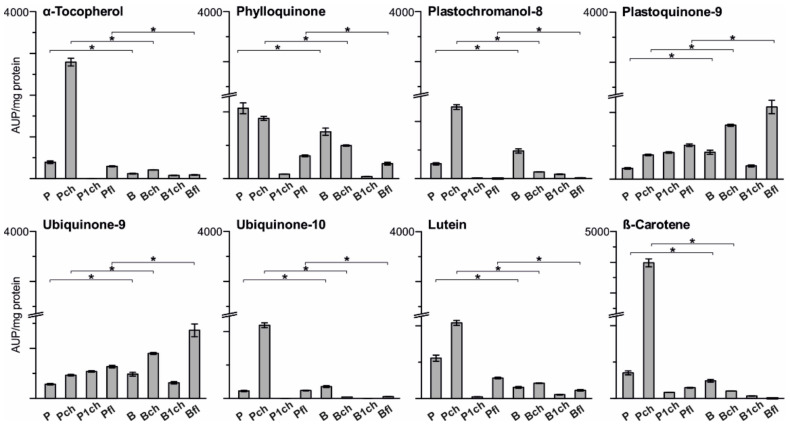
Composition of prenyl lipids and carotenoids isolated from bean (B) and pea (P) plastoglobules (PGs) in control conditions; first leaf (fl) and mature plants, and after the first (1ch) and seventh (ch) day of chilling stress treatment. The composition was presented in mg of total PG protein in precipitated fraction. Pairs of results marked with an asterisk (*) differ significantly at *p* = 0.05 (one-way ANOVA with posthoc Tukey test).

The lipid composition of plastoglobules isolated from pea and bean plants consisted primarily of prenyl lipids and carotenoids as the most abundant detected components (Figure 6). Results were normalised to total PGs protein content in precipitated fractions. Significant differences were observed between the lipid composition of plastoglobules from CS and CT plants in normal growth conditions and after stress treatment. Pea control PGs contained the highest amount of phylloquinone and the lowest detected plastoquinone-9 compared to all remaining PGs fractions, whereas PGs isolated from the CS plant exhibited more significant amounts of plastochromanol-8 and plastoquinone-9 compared to pea PGs (Figure 6). Long-term temperature stress resulted in a significant increase in the content of three major antioxidants: α-tocopherol, plastochromanol-8, plastoquinone-9, and both measured carotenoids: β-carotene and lutein in case of pea chilled PGs (Figure 6). While Bch PGs showed only slightly higher levels of plastoquinone-9, lutein and α-tocopherol were notably lower compared to control conditions than reported in Pch PGs. The levels of phylloquinone, plastochromanol-8, ubiquinone-10 and β-carotene were all decreased under chilling stress in bean PGs, while in pea PGs, no decrease in the content of any of the tested compounds was observed (Figure 6).

Interestingly, two additional quinones were detected in PGs fractions, and based on applied compound standards and lipid retention times were classified as ubiquinone-9 and ubiquinone-10. The presence of ubiquinones in these structures was never previously reported. However, PGs are rich reservoirs of various quinones and contain multiple enzymes involved in their metabolism [1,9,46]. Moreover, one particular enzyme family, the ancient ABC1 atypical kinase family (ABC1K) relevant to coenzyme Q [47,48] and tocopherol metabolism [4], has six protein members present in PGs [49] that could be involved in ubiquinone appearance or synthesis in those lipid structures. In our results, more ubiquinone-9 was detected in bean PGs and stress treated plants (Figure 6, Ubiquinone-9; Bch, Pch) than in PGs from control conditions, while a significantly high level of ubiquinone-10 was reported only in chilled pea PGs (Figure 6). The lipid composition of PGs isolated from bean and pea first leaves was established for the first time. Both Bfl and Pfl contained high amounts of plastoquinone-9 and ubiquinone-9 with a higher amount in bean first leaf PGs. Pea first leaf PGs structures featured greater levels of α-tocopherol, phylloquinone and both carotenoids (Figure 6) compared to bean PGs.

**Figure 7:**
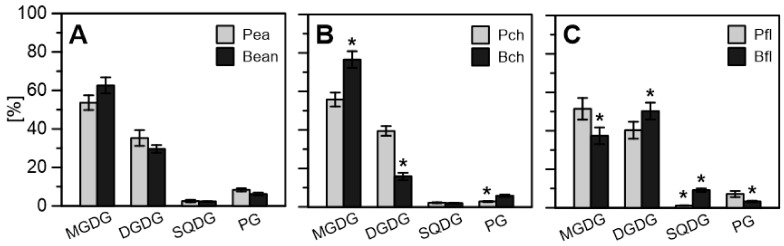
Composition of polar glycerolipids (MGDG, DGDG, SQDG, and PG) in pea and bean plastoglobules (PGs): (**A**) control conditions; (**B**) after the 7th day of chilling stress treatment (ch) and (**C**) in the first leaf (fl). Results marked with an asterisk (*) differ significantly at *p* = 0.05 comparing to control conditions (one-way ANOVA with posthoc Tukey test).

The lipid–protein membrane surrounding the plastoglobule exhibited continuity with the thylakoid membrane, taking the form of a monolayer. It was constituted of the identical main polar lipids: MGDG, DGDG, SQDG and PG (mono-, di- and sulfoquinozyl- diacylglycerol and phosphatidylglycerols). Slight differences in polar lipid content were found (Figure 7A). There was a 15% higher amount of MGDG in bean leaf PGs (63% compared to 53.6% in pea), whereas pea leaves had a higher DGDG level, 36.5% (30% in bean) of the total polar lipid content. Anionic lipids predominated slightly in pea plastoglobules. After chilling, there were no noticeable changes in the lipid composition of pea PGs, while in bean PGs there was a significant increase in the share of MGDG at the expense of DGDG by approx. 10 percentage points. (Figure 7B). In both species, the share of PG decreased. Thus, we found the expression of some distinct chilling stress response strategies in the studied species, which before maturation have a very similar content of MGDG and DGDG (Figure 7C), suggesting some similarity in the early stages of development, probably resulting from current developmental needs. In theory, an increased proportion of the non-bilayer lipid MGDG may translate into greater membrane fluidity [50].

## 3. Discussion

Changes in thylakoid membranes are the main factor in adaptation to chilling stress [25]. Our previous work [29,30,32,51] and recent studies [52] summarise data concerning the structure and arrangement of thylakoid membranes and chlorophyll–protein complexes of pea and bean plants, along with the differences in their responses to chilling stress. As plastoglobules (PGs) are both reservoirs and sinks for components involved in plant responses to chilling stress, we expected that their structure and composition might also vary upon stressor application.

### 3.1. PG Structural Alteration under Chilling Stress

Changes in PGs size, number and osmophilicity under various abiotic stresses have been reported previously in numerous studies [6,11,42,43,53]. According to our data, chilling stress increased PGs size in both analysed plants (Figure 1), although only pea PGs had also increased in number (Figure S1). Additionally, the AFM study revealed that the elasticity of chilling-tolerant (CT) pea PGs did not change significantly under testing conditions, but chilling-sensitive (CS) bean PGs experienced a twofold decrease in Young’s modulus (Figure 2B).

Alteration in the size and amount of PGs from chilled/cold treated plants was reported previously [25,54,55]. The high number of PGs in pea plants may be directly linked with a higher protein and prenyl lipid content. An increase in protein content, but not in the full range of prenyl lipids, can be observed in bean PGs (Figure 4, Figure 5 and Figure 6). Higher content of α-tocopherol and plastochromanol-8 was reported to correlate with number of PGs [41,46,56] and is consistent with the high levels of those compounds detected in chilled pea PGs (Figure 6). The second factor important for increasing the amount of PGs is that FBNs proteins participate in expanding total PGs mass during abiotic stress [4]. As FBNs are mainly involved in building PGs surfaces [33,57,58,59], one may expect a relative increase in FBN content (compared to total protein) with increasing particle size. This correlates with our observation of increasing FBN1 content in chilled bean PGs (Figure 4), which were larger than control ones.

The observed changes in bean PGs elasticity are representative of changes in the particle composition. Although prenyl lipids and carotenoid content (Figure 6) and MGDG and DGDG level (Figure 7) were altered in both pea and bean PGs after chilling, the elasticity changes were observed in bean PGs only. In addition, despite high plastoquinone content, bean PGs were the stiffest in first green leaves and more elastic when exposed to chilling stress (Figure 2B). This suggests that *E* depends more on protein and hydrophobic lipid interior content in PGs, rather than on the abundance of amphiphilic compounds such as prenyl lipids. According to Lundquist et al. [4], PGs morphology can be divided into two zones: a single monolayer periphery filled with prenyl lipids and a PGs interior filled with hydrophobic compounds such as fatty acid phytyl esters (FAPEs) and TAGs (triacylglycerols). Accumulation of TAGs after cold stress treatment was previously reported in leaves of Arabidopsis ecotypes [60]. The ratio of amphiphilic-to-hydrophobic compounds in PGs reflects the PGs’ surface area/volume ratio and, as our results suggest, also implicates their elasticity (Figure 2B), which altered drastically in CS plants after stress treatment.

MGDG, the most abundant thylakoid membrane lipid, may serve as a precursor for TAG synthesis [1,60]. PGs-localised PES1 and -2 proteins containing hydrolase and acetyltransferase domains can use MGDG for fatty acid release and TAG synthesis [1,61]. Results obtained for the polar lipid composition of thylakoids and plastoglobules from both species demonstrate a reverse picture (Figure 7). A lower MGDG abundance in CS plants’ thylakoids was reported in our previous work [52,62] and is attributed to reduced membrane fluidity. However, a higher MGDG content detected in bean PGs suggests a probable redistribution mechanism from thylakoid membranes to PGs interiors, compared to an opposite lipid flow in those of peas (Figure 7B). When chilling treatment was applied, bean thylakoid membranes became more rigid, while the MGDG/DGDG ratio rose [52]. Given our results in the control condition (Figure 7A), we could assume an even higher shift of MGDG towards bean PGs affected by stress and consequently a higher substrate level for TAG synthesis, leading to higher elasticity.

### 3.2. Changes in PG Composition as a Physiological Response to Chilling Stress

Jasmonic acid (JA) signalling has been proposed to play a prominent role in adapting plants to cold stress [27]. JA acts as an upstream signal of the ICE–CBF pathway to positively modulate freezing responses in Arabidopsis, a chilling-resistant plant [63]. Plastoglobules are believed to play a role in jasmonic acid biosynthesis, as they contain JA precursors and four enzymes involved in this mechanism in chloroplasts: LOX 2,-3, AOC and AOS. These enzymes have been reported to be recruited to PGs [1,4,49]. Therefore, the increase observed in the AOS transcript level in the CT leaves of peas after one day of chilling treatment could be seen as a short-term stress signal. On the other hand, bean AOS levels were severely decreased (Figure 5), indicating a lack of response to applied stress conditions in this CS plant.

α-tocopherol could trigger cellular signalling by modulating JA levels [4,26,64]. Recently published materials implicate α-tocopherol deficiency in Arabidopsis mutants in creating a chilling-sensitive phenotype [24,25]. Here, we observed a long-term increase in α-tocopherol content in PGs from chilled plants, especially CT ones (Figure 6). Interestingly, after just one day of chilling stress, α-tocopherol levels drastically decreased. We may then propose that α-tocopherol is used faster than it is synthesised in the short term, and only during long-term adaptation is its level restored and built up. VTE1 is one of the crucial enzymes responsible for α-tocopherol synthesis with confirmed PGs localisation [6,22]. VTE1 transcript levels in pea were downregulated at the beginning of stress treatment, while the AOS level was increased (Figure 5), confirming that α-tocopherol synthesis was inhibited while its use was increased. During the experiment, levels of both transcripts exchanged (Figure 5), and as a result, α-tocopherol content measured in pea PGs after seven days of chilling conditions was much higher than that in bean PGs or those of the pea control (Figure 6). This result points to the previously stated hypothesis [26,64] that changes in the transcript level of these genes led to an increase in the production of tocopherols, resulting in protecting the plant from adverse conditions.

α-tocopherol and plastochromanol-8, whose synthesis is also PGs-localised, are well-documented lipid antioxidants [65,66]. Approximately 50% of synthesised plastochromanol-8 was reported to accumulate in PGs [56], and most probably, its source was plastoquinol-9 (the reduced form of plastoquinone) in the PG-pool [67,68] where VTE1 and NDC1, NADPH-dependent quinone dehydrogenase C1, are also present [69]. Thus, decreased VTE1 levels and lower α-tocopherol and plastochromanol-8 abundance were detected in bean PGs, with higher plastoquinone-9 content than pea PGs (Figure 6), and this reduced the activity of the PGs-localised VTE1 enzyme in bean plants [4,26]. These results and decreased AOS levels in bean PGs indicate that plastoglobules were active “participants” in the chilling stress response. The higher content of plastoquinone in bean PGs (Figure 6) may be related to the sensitivity of bean plants to cold stress. Therefore, bean PGs could be a reservoir of “spare” plastoquinone [1,70]. This compound is an electron carrier from PSII and is credited with antioxidant properties [65]. PGs may serve as a reservoir of antioxidants and replenish the thylakoid pool [56]. It is also tempting to wonder if the spare plastoquinol from bean PGs could replace the plastoquinol in thylakoid membranes, which is degraded irreversibly by ROS in stress conditions [70]. Our previous research described the poor adaptation of beans to low temperatures: stiffening of membranes, disruption of photosynthetic processes, and rearrangement of stacked and unstacked thylakoid areas [51,52], which indicates that such a mechanism could provide additional protection for bean plants against stress conditions.

PGs were also stated to serve as a sink for deposition of excessive amounts of phylloquinone and carotenoids [10,71]. In plants, phylloquinone serves as a light-dependent one-electron carrier within photosystem I from chlorophyll *a* to FeS; thus, it is present mainly in the thylakoid membrane. However, it is not restricted to PSI, as one-third of total chloroplast phylloquinone was found to occur in PGs [9,10,69]. These findings are consistent with previous studies [46,72], because the previously mentioned NDC1 enzyme, located in PGs, is involved in the penultimate step of phylloquinone biosynthesis. Thus, the distribution of this compound into thylakoid membranes may be possible [10,69]. Furthermore, pea PGs under control conditions contained higher levels of detected phylloquinone than control bean PGs (Figure 6), which may reflect their better adaptation to phylloquinone loss in reaction to ROS originating from chilling stress [8].

As a result of chilling, phylloquinone content decreased in both bean and pea PGs, indicating a potential shift of this compound towards thylakoid membranes in stress conditions (Figure 6). Again, the most drastic decrease was found within the short-term response (1d chilling). The phylloquinone content then increased over the following six days, but could not return to the control level. On the other hand, detected lutein and β-carotene contents were higher in CT plant PGs and increased after seven days of applied stress conditions, correlating with the already discussed decrease in short-term response (Figure 6). This result is consistent with our previous work, in which a decrease in carotenoid content, especially β-carotene, in chilled pea thylakoids was observed, suggesting a “spare” pool could accumulate in PGs (Figure 6) [30,52]. Moreover, maintaining the thylakoid membrane’s optimal fluidity under temperature stress is linked with the balance between free hydrophobic carotenoids: xanthophylls and β-carotene. Therefore, storage of excess amounts of carotenoids (e.g., tocopherol and plastoquinone) in PGs of a CT plant might be an example of thylakoid–PG cooperation in coping with stress conditions.

### 3.3. PGs Structure and Composition Development from First Green Leaves

We saw a significant difference in the number, size and osmiophilicity (electron density) of plastoglobules in our pea and bean plants’ plastids during their biogenesis [32]. In pea plants, the number of plastoglobules was high in etiolated seedlings. They often lay very close to the prolamellar body (PLB) or even sometimes inside the organised PLB structure [73]. On the other hand, they were of lower electron density and generally larger (Figure 1) than those detected in bean first leaves. Additionally, after illumination, the plastoglobules appeared scattered, less “osmiophilic”, and smaller in mature pea and bean plants (Figure 1). Results were consistent with previous reports indicating that during de-etiolation, the abundance of prolamellar bodies (PLBs) and PGs decreased simultaneously with thylakoid formation [1,32]. This phenomenon suggests PGs play a role in thylakoid development, probably by providing prenylquinones and TAG for membrane lipid synthesis [74].

Our past results showed that the transformation of etioplasts into chloroplasts and the PLB unravelling process occurred divergently in pea and bean plants (faster in the bean plants) [32], indicating a different role for PGs in this transition. Furthermore, results obtained by Ytterberg [6] and Blomqvist [75] showed that the protein composition of PGs from rice (*Oryza sativa*) etiolated seedlings and PLBs from dark-grown wheat (*Tritium* spp.) leaves was not identical and differed extensively from that of chloroplast PGs [6]. The lack of a protein signal in immunodetection analysis (not shown) of FBN1 (PGL35), combined with severely reduced transcript levels (Figure 5A) in PGs of both pea and bean first leaves, confirms this statement. Additionally, our lipid composition results (Figure 6) show the presence of carotenoids and prenylquinones in PGs isolated from pea and bean first leaves, supporting their presumed function in thylakoid formation [1] as suppliers of prenylquinones and carotenoids for developing and stabilizing photosynthetic membranes. A similar function may be proved by the unified content of MGDG and DGDG in the membrane leaflets of PGs isolated from the first leaves of both plants (Figure 7C). This suggests some more universal mechanism or need for specific content of membrane lipids at this stage of development [76]. Galactolipids provide thylakoids with adequate fluidity or stiffness and allow for the preservation of a high protein-to-lipid ratio and large protein-pigment complexes within the membrane.

Interestingly, plastoquinone-9 and ubiquinone-9 were the most abundant prenyl lipids detected in first leaf PGs. The presence of ubiquinone compounds in PGs was never previously observed, while plastoquinone, with its robust antioxidant ability, could be stored inside first leaf PGs and redistributed under stress conditions to detoxify ROS. Additionally, proteins present in PGs from the fibrillin family were proposed to play a significant role in regulating plastoglobular lipid content [77]. Specifically, it was demonstrated that FBN4 is strongly linked with prenylquinone accumulation in PGs [33]. Thus, the lack of the FBN1 protein in our results may be compensated for by other fibrillin family proteins located in PGs [1,49], most likely FBN4, as the plastoquinone level in bean and pea first leaves PGs were high (Figure 6). The Appletree *fbn4* knockdown mutant exhibited a phenotype sensitive to various abiotic stresses due to decreased plastoquinone content in PGs. In contrast, its leaf content was comparable to those in control plants, indicating that storage of this antioxidant compound in PGs could be a defence mechanism [33].

## 4. Materials and Methods

### 4.1. Plant Material and Stress Treatment

Seeds of *P. sativum* and *Ph. coccineus* were surface sterilised in chlorine and placed into 3 l perlite-containing pots in a climate room (22 °C/20 °C day/night temperature), at active photosynthetic radiation (PAR) of 200 μmol photons m^−2^ s^−1^ during a 16 h photoperiod and relative humidity of 60–70%. Plants were fertilised with full Knopp’s nutrient solution, as we described previously [31,51,52].

For the chilling treatment, plants were exposed to low temperatures (5 °C) at night, while daytime temperature in the climate room was optimal for these plant species (22 °C). Chilled leaves were investigated after one and seven days. Leaves were collected after 30 min of light exposure. The first leaves indicate the first leaves appearing above the ground.

### 4.2. Isolation and Purification of Plastoglobules

The whole purification process was performed at 4 °C to preserve the chloroplasts’ integrity and avoid protein degradation. The procedure started with isolating intact chloroplasts by centrifugation on a Percoll gradient (Percoll Plus, Cytiva, Marlborough, MA, USA). Plastoglobules were then separated from plastids membranes by flotation on a sucrose gradient. The Besagni et al., protocols were used with slight modifications, designed to adapt to insulation consisting of more material than described in the protocol [78]. Finally, the purity of the plastoglobule fraction was verified by immunoblotting (Figure S2).

### 4.3. Protein Concentration Determination

Isolated plastoglobules in sucrose fractions were purified and concentrated in TrE buffer (5 mM Tricine-KOH, pH 7.5, 0.2 mM EDTA, and 0.2 mM dithiothreitol) using disposable ultrafiltration devices (Vivaspin 500 Concentrators, Sartorius Stedim Biotech GmbH, Goettingen, Germany). First, 400 µL of deterged fractions were precipitated as described previously [78], with slight modification; obtained PG proteins were resuspended in 10 µL in TrE buffer, and the BCA protein assay kit for low concentrations (ab207002, Abcam, Cambridge, UK) was used to quantify the protein content. The assay was performed according to the manufacturer’s instructions. An equal volume of every sample was added to the BCA working reagents, and the reactions were incubated for 120 min at 37 °C. Absorbance was read at 562 nm on a NanoDrop 2000/2000c Spectrophotometer (Thermo Fisher Scientific Inc., Waltham, MA, USA). Protein concentrations were calculated using bovine serum albumin (BSA) standards and a four-parameter logistic curve using NanoDrop BCA PROTEIN software (v. 1.6, Thermo Fisher Scientific Inc., Waltham, MA, USA).

### 4.4. SDS-PAGE and Immunoblot Analysis

Every sample was suspended in Laemmli buffer with slight modifications—6M urea and 150 mM DTT were added for better protein denaturation. Samples containing 30 µL of plastoglobule proteins were loaded into each well containing stacking gel. Standard SDS-PAGE conditions were used as described previously [51].

Proteins separated by SDS-PAGE were detected on a PVDF membrane (Merck Millipore, Burlington, MA, USA) using 1:1000 *v*/*v* primary rabbit antibody anti-plastoglobulin 35 (PGL35/FBN1a AS06 116, Agrisera AB, Vännäs, Sweden). Subsequently, the ECL Detection System (Bio-Rad Laboratories Inc., Hercules, CA, USA) was used to detect secondary anti-rabbit antibody conjugated to horseradish peroxidase, according to the manufacturer’s protocols (Bio-Rad Laboratories Inc., Hercules, CA, USA). Finally, Image Studio Digits Software was used to analyse the density of identified bands (Li-COR Biosciences, Lincoln, NE, USA).

### 4.5. Electron Microscopy and CLSM

The extracted plastoglobule fraction was purified from the sucrose gradient using Vivaspin Concentrators (Vivaspin 500 100kDa MWCO, Sartorius Stedim Biotech GmbH, Goettingen, Germany) and then stained with 0.5% OsO_4_ [33] and placed on a 400 mesh formvar-coated copper grid, stabilised with light layer carbon film (Polyscience Europe GmbH, Eppelheim, Germany) and analysed using a JEM 1400 (JEOL Co., Akishima, Japan) transmission electron microscope (TEM).

### 4.6. Atomic Force Microscopy

AFM measurements were performed with an Agilent 5500 (Agilent Technologies Inc., Santa Clara, CA, USA) microscope, working in contact and tapping modes, under the control of PicoView software (Agilent Technologies Inc., Santa Clara, CA, USA). Samples were imaged in a liquid cell, and SNL probes (Bruker Corp., Billerica, MA, USA) with nominal k = 0.35 N/m were used. For elasticity determination, the k constant of each probe was measured with a built-in Agilent Thermal-K setup. Plastoglobule samples were immobilised on freshly cleaved mica, with poly-L-lysine used as stabilising matrix.

Images were recorded at 1 ln/s and resolution 512 × 512, with minimal possible force applied. Surface probing for elasticity determination was done with a built-in plugin for PicoView software, recording and analysing force-distance curves. Usually, a resolution of 32 × 32 was used for probing a chosen region of approximately 2 μm × 2 μm. Images were processed with Gwyddion 2.49 software [79,80].

### 4.7. FTIR Analysis

Suspensions of isolated plastoglobules were resuspended in a D_2_O-based 20 mM Hepes–NaOH (pH 7.0) buffer containing 330 mM sorbitol and then centrifuged at 7200× *g* for 10 min at 4 °C. This step was repeated three times to replace H_2_O based buffers with D_2_O ones. Fourier-transform infrared (FTIR) spectra were recorded with a Bruker Vector 33 spectrometer equipped with a horizontal attenuated total reflection (ATR) cell and a Nicolet iS50 FTIR spectrometer (Thermo Fisher Scientific, Waltham, MA, USA) equipped with a single reflection diamond ATR cell. Plastoglobules were deposited on a ZnSe or diamond crystal (Nicolet) and spectra were recorded at a spectral resolution of 0.6 cm^−1^. The buffer without plastoglobules was used as a control. For each background and sample spectrum, 25 interferograms were averaged and Fourier transformed. Data analysis was carried out with Grams/AI 8.0 Spectroscopy Software (Thermo Electron Corp., Waltham, MA, USA).

### 4.8. Transcriptome Analysis

Total RNA was extracted with Ribospin^TM^ Plant Total RNA Purification kit (GeneAll Biotechnology Co., Ltd., Seoul, Korea), according to the manufacturer’s instructions, using DNase digestion, and then eluted with 50 μL of water. Directly following isolation, RNA quality was checked with a NanoDrop spectrophotometer (Thermo Fisher Scientific Inc., Waltham, MA, USA). RNA was stored at −80 °C and thawed only shortly before the experiment. An appropriate set of reference genes was created for each plant, simultaneously reflecting the housekeeping genes unchanged during ontogenesis and the effects of abiotic stress. Tub1 and PP2A were selected for pea plants and Act11 and Tub8 for bean plants.

Primer sequences for all genes were designed using Primer-BLAST (NCBI) and checked for specificity by BLAST searching the RefSeq RNA database (Table S1).

Expression analysis was conducted by quantitative PCR in a MyGo Pro Real-Time PCR thermocycler (IT-IS INTERNATIONAL LTD., Stokesley, UK) using SensiFAST One-Step MasterMix for SYBR Green No ROX (Bioline, Meridian Bioscience Inc., Cincinnati, OH, USA) with the recommended thermal profile (45 cycles). Following amplification, a melt curve was performed in the 60–95 °C range with 0.5 °C steps.

Each sample’s relative gene expression was calculated with MyGoPro analysis software (v. 3.3, IT-IS INTERNATIONAL LTD., Stokesley, UK) and scaled to the calibrator sample (usually the control plant). Intra-assay variation was evaluated by calculating SD errors of arithmetic means of sample replicates.

### 4.9. Lipidomic Analysis of Plastoglobules

Extraction of polar lipids from 200 µL of isolated PGs fraction was carried out using a slightly modified Folch method (addition of chloroform-methanol 1:2 (*v*/*v*) mixture). The resulting mixture was shaken and centrifuged. For complete recovery, the pellet was re-extracted with methanol:chloroform:water 2:1:1 (*v*/*v*/*v*). The combined extract was dissolved in chloroform and 0.2 M KCl. The lower organic phase was collected, evaporated, and used in extraction from solid phase (SPE) after adsorption on diatomite. The eluate was then evaporated to dryness using an Heidolph Laborotary 4000 evaporator at 50 °C, and dissolved in a chloroform:methanol 1:1 (*v*/*v*) mixture.

The sample was injected into an HPLC Discovery™ Supelco RP Amide 2.5 × 150 mm C-16 column in an HPLC/ESI-MS system (WATERS 600 coupled with WATERS MICROMASS ZQ). Elution was carried out by a gradient of solvents: water followed by methanol-acetonitrile 7:3 (by volume) in 120 min (this method is a modification of Gil et al.’s method [81]). Quantitative analysis was performed based on areas under the spectrum calculated using MassLynx v. 3.5 software (v. 4.1, Waters Corp., Milford, MA, USA).

### 4.10. Prenyl Lipid and Carotenoid Extraction

An exact volume (300 µL) of isolated plastoglobule fractions with measured protein concentrations (mg of plastoglobule protein in precipitated fractions) was used for analysis. Lipids were extracted as described previously in [82] with slight modifications; volumes of solvents used in this step were halved and adjusted to low fraction amounts. The HPLC analysis of prenyl lipids and carotenoids was carried out using Sztatelman et al.’s BMC Plant Biology (2015) [83]. Aliquots of 40 µL of methanol lipid extracts were loaded with a loop onto a C-18 column (Bionacom Velocity, 5 microns, 4.6 × 250 mm, BIONACOM LTD, Coventry, UK). The lipids were identified using a UV detector (290 nm for prenyl lipids, 460 nm for carotenoids) integrated with a Dionex ICS-3000 chromatograph (Thermo Fisher Scientific Inc., Waltham, MA, USA). Pigments and prenyl lipids were identified by retention time and characteristic UV absorption spectrum and compared to applied standards (Figure S3). The α–tocopherol, phylloquinone (K), coenzyme Q9 and coenzyme Q10 (ubiquinone-9 and ubiquinone-10, respectively) standards of HPLC grade (≥99.5%) were obtained from Sigma-Aldrich (Sigma Aldrich Inc., Saint Louis, MO, USA). Chromatogram analysis and peak areas were determined using Chromoleon software (Thermo Fisher Scientific Inc., Waltham, MA, USA).

## 5. Conclusions

In conclusion, we confirmed that changes occur in the composition and structure of plastoglobules after chilling stress. These alterations were not universal; we observed differences between the CT pea and the CS bean. The PGs reaction to lower temperature seemed to be dynamic and occurred after short exposure to chilling stress. A typical physiological response of mature leaves to several days of chilling was an increase in PG size or in components such as plastoquinone-9, and a decrease in phylloquinone levels. In answer to stress, it seems likely that PGs supply antioxidants to the thylakoid membranes with which they are in close contact. At an earlier stage of leaf development, the structure and composition of PGs were very similar for both studied plants. This suggests the occurrence of developmental demand and a universal phenomenon that is altered during maturation.

The bottom line is that these inconspicuous protein–lipid particles may have more essential functions in photosynthetic membrane dynamics in response to chilling stress than previously thought.

## Data Availability

The data presented in this study are available on request from the corresponding author.

## Supplementary Materials

The following are available online at https://www.mdpi.com/article/10.3390/ijms222111895/s1.

## Abbreviations

CS	Chilling-sensitive
CT	Chilling-tolerant
PG	Phosphatidylglicerol
PGs	Plastoglobules
