# Supplementary material for: Bean and Pea Plastoglobules Change in Response to Chilling Stress
Source: Int J Mol Sci. 2021 Nov 2;22(21):11895. doi: 10.3390/ijms222111895 (PMC8584975; doi:10.3390/ijms222111895)
Supplement: Supplementary file 1 [file ijms-22-11895-s001.zip › ijms-1403336-supplementary.pdf]

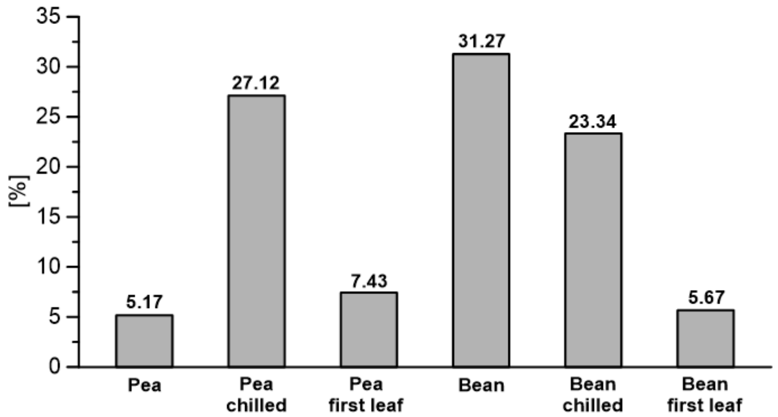

Figure S1. The amount of plastoglobules measured from TEM images,presented as percentage distribution from all analyzed variants, total counts= 2400 PGs.

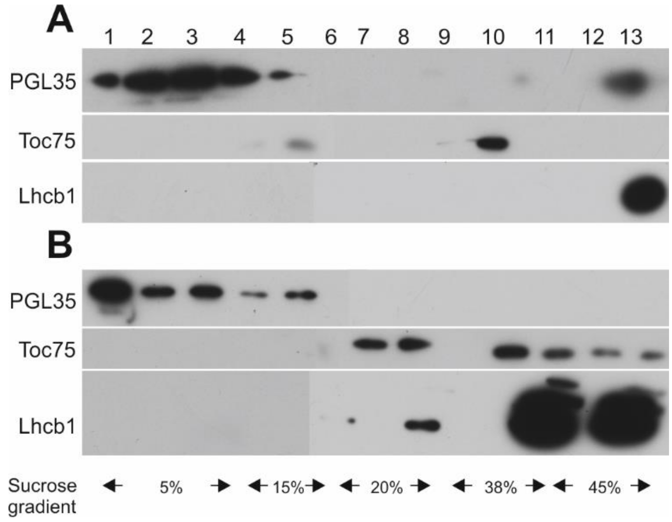

Figure S2. Immunoblot analysis of chloroplast membrane fractions isolated from pea (*Pisum sativum*) and bean (*Phaseolus coccineus*) plants in control conditions. Chloroplast membranes were separated by ultracentrifugation on a sucrose gradient. Fractions of 2 mL were collected from the top (fraction 1 in 5% sucrose) to the bottom (fraction 13 in 45% sucrose) of the sucrose gradient. Proteins from 400 μL aliquots of the even fractions 1-13 were precipitated, separated by SDS-PAGE, and transferred to a PVDF membrane. The membrane was sequentially probed with antibodies raised against membrane marker proteins: PGL35 (plastoglobule protein), Toc75 (outer envelope membrane protein), and Lhcb1 (thylakoid protein). Gels were scanned using C-DiGit Blot Scanner (Li-COR Biosciences, Lincoln, Nebraska, USA) in a resolution of 300 dpi and TIFF format.

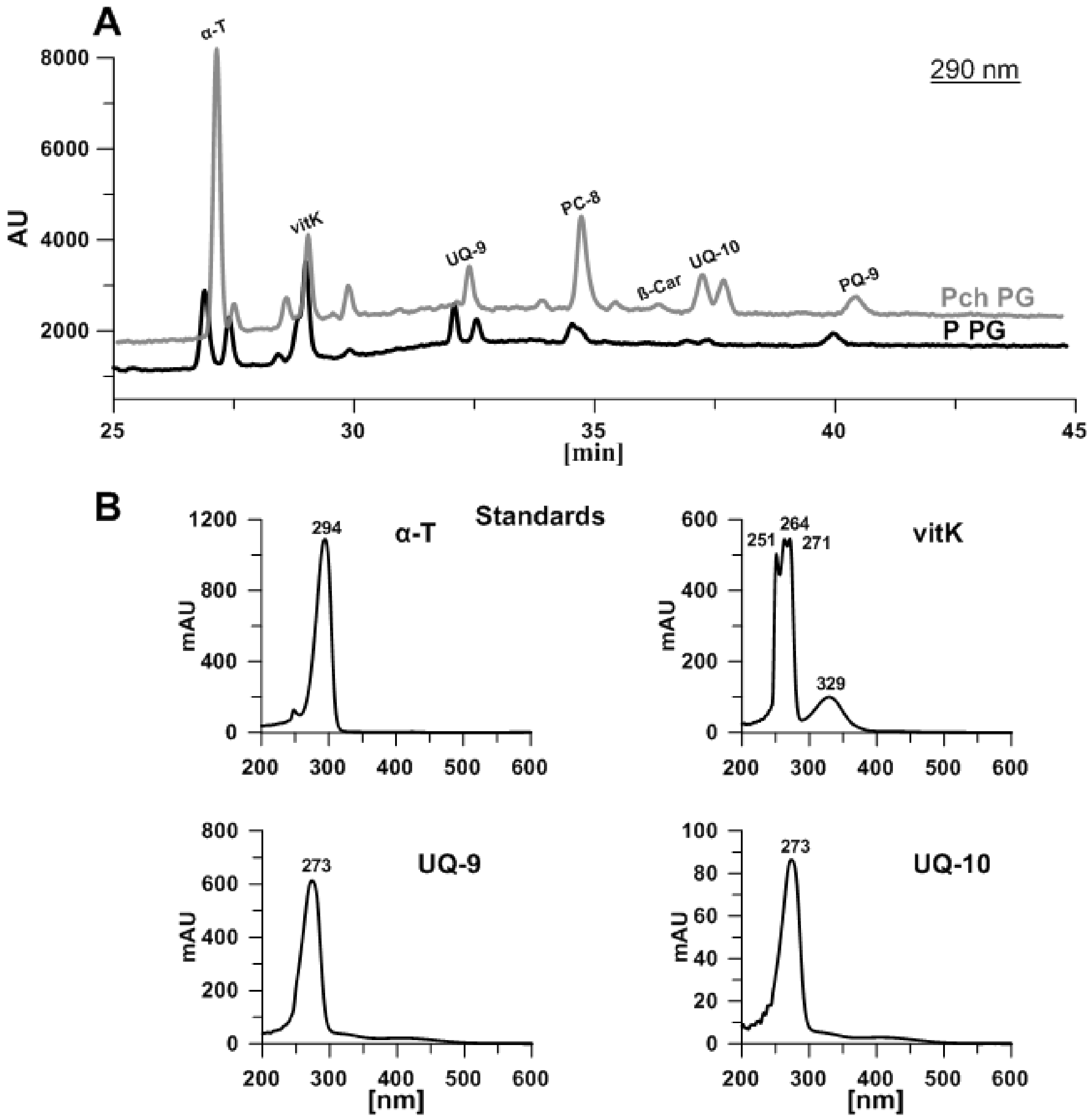

Figure S3. Prenyllipid and carotenoid profilling in PGs. (A) Identification of peaks present in chromatograms of PG extracts from .pea control and chilled plants as an example. Abundant peaks were indicated by abbreviations as follows: α-T- α-Tocopherol, vitK- Phylloquinone, UQ-9- Ubiquinone-9, PC-8- Plastochromanol-8, ß-Car- ß-carotene, UQ-10- Ubiquinone-10, PQ-9- Plastoquinone-9. (B) Pigments and prenyllipids were identified by retention time [1,2] characteristic UV absorption spectrum [3] and compared to applied standards. The α–tocopherol (α-T), phylloquinone (vitK), coenzyme Q_9 and coenzyme Q_{10} (ubiquinone-9 and ubiquinone-10, respectively) standards of HPLC grade (≥99.5%) were obtained from Sigma-Aldrich.

Table S1. List of primers used in this research.

Name	Forward sequence 5'→ 3'	Reverse sequence 5'→ 3'
**PP2A** *(pea)*	TGATCAAGTGAAGATGTCCAGCTA	GCAAAGCAGGATCTCAACAA-TAACT
**TUB1** *(pea)*	ATTCTCTGAGGCTCGTGAGG	AAAAGCACATTGCACAACACAAA
**PGL35** *(pea)*	TGAGGTGTCCGAGTTTGTGG	TGTGTACAGCAGCACCCAAT
**AOS** *(pea)*	TGACTCCGGCGATCAAACAG	AGAATTGGACCGAGCTGACG
**VTE1** *(pea)*	TTCATGGTGTATTGAGCACGC	ACTGCATGCGGAAGCTCAAG
**Act11** *(bean)*	CTGGCCTACATTGCCCTTGA	CTGTCCATCAGGCAACTCGT
**Tub8** *(bean)*	ATGAGGAGGAAATTGCACCA	TTACACCACTCGTTCAAAGCC
**PGL35** *(bean)*	CCGTCCCCGTCTCCTAAGAA	GGGTTGACACTGTCTTTCCGA
**AOS** *(bean)*	CACGCGTCCGAGTTACTTTT	GAGGCTCCCAGAACTTGCTT
**VTE1** *(bean)*	AAGAGGGTGGGGATAACAGC	GATGCCAGATCCACCCAGG

References

1. Martinis, J.; Kessler, F.; Glauser, G. A novel method for prenylquinone profiling in plant tissues by ultra-high pressure liquid chromatography-mass spectrometry. *Plant Methods* **2011**, *7*, doi:10.1186/1746-4811-7-23.
2. Kessler, F.; Glauser, G. Prenylquinone Profiling in Whole Leaves and Chloroplast Subfractions. *Plant Isoprenoids: Methods and Protocols* **2014**, *1153*, 213-226, doi:10.1007/978-1-4939-0606-2_15.
3. Fraser, P.D.; Pinto, M.E.S.; Holloway, D.E.; Bramley, P.M. Application of high-performance liquid chromatography with photodiode array detection to the metabolic profiling of plant isoprenoids. *Plant Journal* **2000**, *24*, 551-558, doi:10.1111/j.1365-313X.2000.00896.x.
